# The *diaphanous* Gene of Drosophila Interacts Antagonistically with *multiple wing hairs* and Plays a Key Role in Wing Hair Morphogenesis

**DOI:** 10.1371/journal.pone.0115623

**Published:** 2015-03-02

**Authors:** Qiuheng Lu, Paul N. Adler

**Affiliations:** 1 Biology Department, University of Virginia, Charlottesville, Virginia, United States of America; 2 Cell Biology Department, University of Virginia, Charlottesville, Virginia, United States of America; University of Dayton, UNITED STATES

## Abstract

The Drosophila wing is covered by an array of distally pointing hairs that has served as a key model system for studying planar cell polarity (PCP). The adult cuticular hairs are formed in the pupae from cell extensions that contain extensive actin filaments and microtubules. The importance of the actin cytoskeleton for hair growth and morphogenesis is clear from the wide range of phenotypes seen in mutations in well-known actin regulators. Formin proteins promote the formation of long actin filaments of the sort thought to be important for hair growth. We report here that the formin encoding *diaphanous* (*dia*) gene plays a key role in hair morphogenesis. Both loss of function mutations and the expression of a constitutively active Dia led to cells forming both morphologically abnormal hairs and multiple hairs. The conserved *frizzled* (*fz*)/*starry night* (*stan*) PCP pathway functions to restrict hair initiation and activation of the cytoskeleton to the distal most part of wing cells. It also ensures the formation of a single hair per cell. Our data suggest that the localized inhibition of Dia activity may be part of this mechanism. We found the expression of constitutively active Dia greatly expands the region for activation of the cytoskeleton and that *dia* functions antagonistically with *multiple wing hairs* (*mwh*), the most downstream member of the *fz/stan* pathway. Further we established that purified fragments of Dia and Mwh could be co-immunoprecipitated suggesting the genetic interaction could reflect a direct physical interaction.

## Introduction

The Drosophila wing is covered with an array of distally pointing hairs that defines the planar cell polarity (PCP) of the tissue [1,2]. Genetic studies led to the identification of the *frizzled* (*fz*)/*starry night* (*stan*) pathway as the key regulator of PCP [3]. Further studies have shown that this pathway functions in other body regions and is conserved in essentially all animals to regulate PCP [2,4]. A characteristic of the system is that protein members of the pathway accumulate on either the distal, proximal or both of these sides of pupal wing cells [1,2]. A second pathway, the *ds/ft* pathway also regulates PCP [5–8]. In the wing and the eye *ds/ft* is generally thought to function upstream of the *fz* pathway [9–11] and there is evidence that it does so by regulating the orientation of the microtubule cytoskeleton that is used for the directed trafficking of PCP proteins [12–14]. Although the microtubule cytoskeleton has received more attention with regard to the asymmetric accumulation of PCP proteins it is worth noting that two genes that encode proteins that promote actin filament depolymerization, *twinstar* (*tsr-*cofilin) [15] and *flare* (*flr-*AIP1) [16] have been found to be essential for the generation of wing PCP and the distinctive protein accumulation pattern.

Mutations in genes that regulate the actin cytoskeleton often result in abnormal hairs. Among the good examples are the bundling proteins *singed* (fascin) [17] and *forked* (forked) [18,19] that result in twisted and bent hairs and the myosins *crinkled* (myosin VIIa) [20] and *zipper* (myosin II) [21,22] which result in short, split and multipled hairs. Mutations in the small GTPases *Rho1*, *Dcdc-42* and the effector Rho kinase (*Drok*) also result in short, split and multipled hairs [23–25]. Perhaps the most extreme hair phenotypes are associated with *tsr* and *flr* mutations [15,16]. Mutations in the *slingshot* phosphatase that dephosphorylates and activates cofilin also produces hair morphology phenotypes [26]. Drugs that antagonize the actin cytoskeleton also result in abnormal hair morphology providing further evidence for the importance of actin in hair growth [27]. The growing hair is likely to contain long actin filaments [28]. Formins are known to promote the formation of long linear actin filaments [29,30] and hence are strong candidates for having a role in hair morphogenesis. Indeed, one formin, *diaphanous* (*dia*) is known to be important for the morphogenesis of denticles in the embryo [31]. Denticles are in some ways similar to hairs/trichomes and many, but not all genes show similar mutant phenotypes in both [32]. We explored the role of the Drosophila formin genes in wing hair development and found *dia* to be a key gene. Both loss and gain of function mutations result in dramatic abnormalities in hair morphology. We also established that *dia* also plays an important role in the morphogenesis of sensory bristles, a another polarized cell type where linear actin filaments are prominent and thought to be important [33,34].

Growing hairs also contain centrally localized microtubules that are likely to be important for hair growth [23,27,35]. Indeed, the application of drugs or the expression of transgenes that antagonize the microtubule cytoskeleton results in the formation of multiple hairs [13,27]. There is however, little loss of function genetic data establishing the importance of the microtubule cytoskeleton in hair outgrowth.

The *fz/stan* pathway regulates wing PCP by restricting the activation of the cytoskeleton that drives hair morphogenesis to the distal most part of the cell [3]. The *multiple wing hairs* (*mwh*) gene is the most downstream member of the *fz* pathway and hence is a strong candidate for mediating at least part of this restriction [3,36,37]. Mwh accumulates on the proximal side of wing cells prior to hair morphogenesis and later it is also found in the growing hair [36,37]. *mwh* mutations result in most wing cells forming 3 or more hairs with aberrant polarity at abnormal locations along cell periphery [3,36,37]. A variety of data suggests that Mwh acts as an inhibitor of the actin cytoskeleton. For example, the high level over expression of *mwh* leads to a delay in hair initiation, loss of function mutant cells form extra hairs and ectopic actin filaments and the expression of *mwh* in cultured cells leads to actin phenotypes [36,37].

The sequence of the Mwh protein suggests a possible mechanism for mediating PCP control of the actin cytoskeleton. The amino terminal half shows similarity to the same region in Diaphanous family formins [36,37]. This region contains two sequence motifs: a GTPase binding domain (GBD) and a *formin* homology 3 domain (FH3) [38,39]. The GBD-FH3 domain was divided into 3 structural domains: a GBD domain (which is smaller than the region originally identified as the GBD), a *diaphanous* inhibitory domain (DID), and a dimerization domain (DD) [29,40–44]. Dia activity is inhibited by the intramolecular binding of the C terminal DAD (diaphanous autoregulatory domain) to the DID [42,45]. In this conformation the carboxy terminal FH1 and FH2 domains cannot promote actin polymerization. A conformational change occurs with the binding of Rho-GTP and this relieves the inhibition. Previous data from our lab suggested that Mwh was also activated by Rho-GTP binding implying that Mwh also exists in an auto inhibited state [25]. However, the Mwh protein does not contain FH1 and FH2 domains, and is not expected to be able to promote actin polymerization like true formins. The existence of a dimerization domain within the similarity region of Mwh and Dia suggests that Mwh might heterodimerize with Dia and inhibit Dia function.

We report here that the expression of a constitutively active Dia (CA-Dia) in pupal wing cells greatly increases the area where F-actin accumulation is seen at the start of hair formation. Thus, it appears that the presence of CA-Dia antagonizes the ability of the *fz/stan* pathway to restrict the activation of the cytoskeleton to a small distal region. We also carried out genetic studies that show *mwh* and *dia* act antagonistically in wing hair development and that purified fragments of these two proteins interact in vitro. Surprisingly this physical interaction was not based on the DD domains that we suspected would mediate the dimerization. These data suggest that one possible mechanism by which the *fz/stan* pathway regulates wing PCP is by the direct inhibition of Dia by Mwh.

## Results

### The role of *dia* in wing hair morphogenesis

Dia and other formins are known to promote the polymerization of long actin filaments, which are thought to be important for hair morphogenesis [28,46]. All of the six Drosphila formins are expressed in pupal wings and none show an increase in expression at the time of hair formation hence expression pattern did not point to any candidates [47]. To determine if any formin played a non-redundant role in wing hair morphogenesis we used transgene mediated RNAi driven by *ptc-Gal4* to knock down levels of the Drosophila formins in the wing [48]. We tested: *dia*, *cappuccino*, *DAAM*, *formin 3*, *CG32138* and *Formin homology 2 domain containing ortholog* (*Fhos*). Of these only *dia* showed a substantial wing hair phenotype consistent with a possible role as a downstream target of the *fz* pathway and/or a key role in hair morphogenesis. A hint of a mutant phenotype was seen for *CG32138*, but its weakness and weak penetrance lead us to give it a low priority for further study. No phenotype was seen in the knockdowns of the other formins. In a separate set of experiments we also used transgene mediated RNAi knockdowns to determine if Arp2/3 complex proteins, which promote the formation of branched actin networks [49,50] had a role in hair morphogenesis. We found that knocking down *p16ARC* (*CG9881*), *Arp11* (*CG12235*), *Arc-p34* (*CG10954*), *Arpc3A* (*CG4560*), *Arpc3B* (*CG8936*) and *Arp14D* (*CG9901*) using *UAS-dicer2; ptc-Gal4* all produced a similar set of phenotypes with varying severity (S1 Fig.). The most common was a swollen hair base (S1 Fig.). This was seen in essentially all hairs in the proximal part of the *ptc* domain. We also observed double hair cells of normal polarity (black arrow), branched hairs (red arrowhead), polyploid cells and decreased alignment of neighboring hairs (red arrows). We also failed to see a phenotype with a knock down of *spire*, another actin regulator.

**Fig 1:**
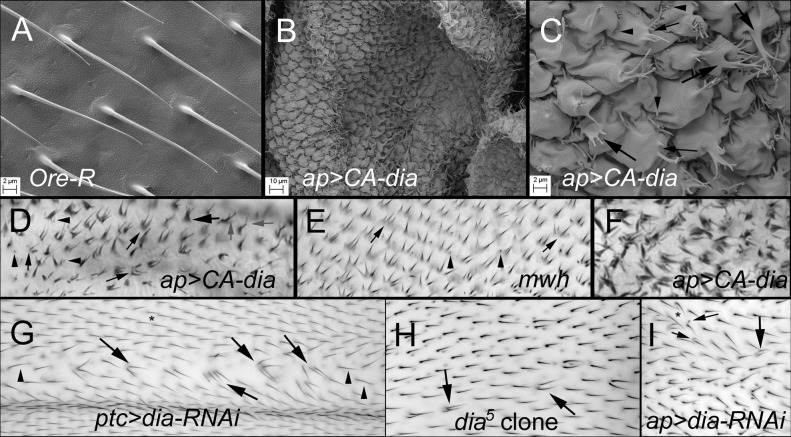
Wing hair phenotypes of both loss of function and gain of function *dia* mutations. A-C are SEM images and D-I are bright field images. A. Oregon-R, B. An *ap>CA-dia* wing at a lower magnification to see the folded wing phenotype. C. An *ap>CA-dia* wing shown at the same magnification as the wild type in A. Note the bumpy and folded cells and that hairs do not show a consistent polarity. Large arrows point to cells with thick hairs that contain a number of thin distal projections, some of which are branched. Small arrows point to cells that contain more than one independent hair. Arrowheads point to very small hairs/projections. D. *ap>CA-dia*. In all bright field images distal is to the right and proximal to the left. Large arrow points to a hair with a thick proximal region and 3 thin distal projections. The small arrows point to cells that formed multiple independent hairs. Grey arrows point to cells that formed branched hairs. The arrowheads point to very small hairs. Many of the cells show abnormal polarity (polarity should be pointing toward the right). E. A *mwh* ^*1*^ wing where hairs show abnormal polarity (i.e. they do not point to the right (distally). The arrows point to cells that formed multiple hairs. The arrowheads point to very small hairs. F. *ap>CA-dia* wing showing a light micrograph of the very strong phenotype shown in SEM in C. The pattern of hairs is too abnormal to easily interpret. G. *ptc>dia-RNAi*. The arrows point to polyploid cells in the region where *ptc-Gal4* drives the highest level of expression that formed multiple hairs. The arrowheads point to polyploid cells that formed a single hair. Note the hairs are longer than the hairs outside of the region where *ptc* drives the highest level of expression (marked by the an asterisk). H. A wing region containing unmarked *dia* ^*5*^ clones that produced several polyploid cells that form more than one hair (arrows). Such an abundance of clone cells was unusual in our experiments. I. A wing region of a *ap>dia-RNAi* fly that contains diploid multiple hair cells (large arrow). Note that in contrast to G and H the multiple hair cell is not larger than the neighboring cells. Some of the cells form very small hairs (small arrows). Some of the hairs show a weak polarity phenotype (asterisk).

The predominant phenotype seen with the knock down (kd) of *dia* was polyploid cells (Fig. 1G, arrows, arrowheads). When we used *ptc-Gal4* to drive the kd this was seen all wings but only in a thin band (~3 cells wide) down the center of the wing where *ptc-Gal4* drives expression at the highest level (Fig. 1G). Polyploid cells were not surprising as *dia* is known to have an important role in cytokinesis [51,52]. Greater than 95% of the cells in this band appeared to be polyploid. Twenty six percent (0.04sd) formed multiple hairs (Fig. 1G, arrows) and 70% (0.04sd) formed a single hair that was longer than normal (Fig. 1G, arrowheads). Both types of cells/hairs are easily recognized as being polyploid due to their larger size and the greater distance between neighboring cells hairs [53]. It is worth noting that the *fz* pathway is functional in polyploid wing cells [53] but it is not able to scale to force the formation of only a single hair in these large cells. To minimize the formation of polyploid cells and cell death [51] we used temperature shifts and a temperature sensitive Gal80 to limit the period of time when *dia* expression was knocked down to after cell division had ceased in the wing [54]. Under favorable conditions when we used *ap-Gal4* as a driver we found multiple hair cells (mhc) that did not appear to be polyploid (Fig. 1I, large arrows) and some regions where hairs showed abnormal polarity (Fig. 1I, asterisks). The frequency of these hairs was variable from fly to fly perhaps due to slight variation in the timing of the temperature shift. In addition to the mhc we also saw hair morphology abnormalities including split hairs and short single hairs (Fig. 1I, small arrows). Chemical inhibitors of actin polymerization such as cytochalasin-D produce similar hair morphology phenotypes [27].

**Fig 2:**
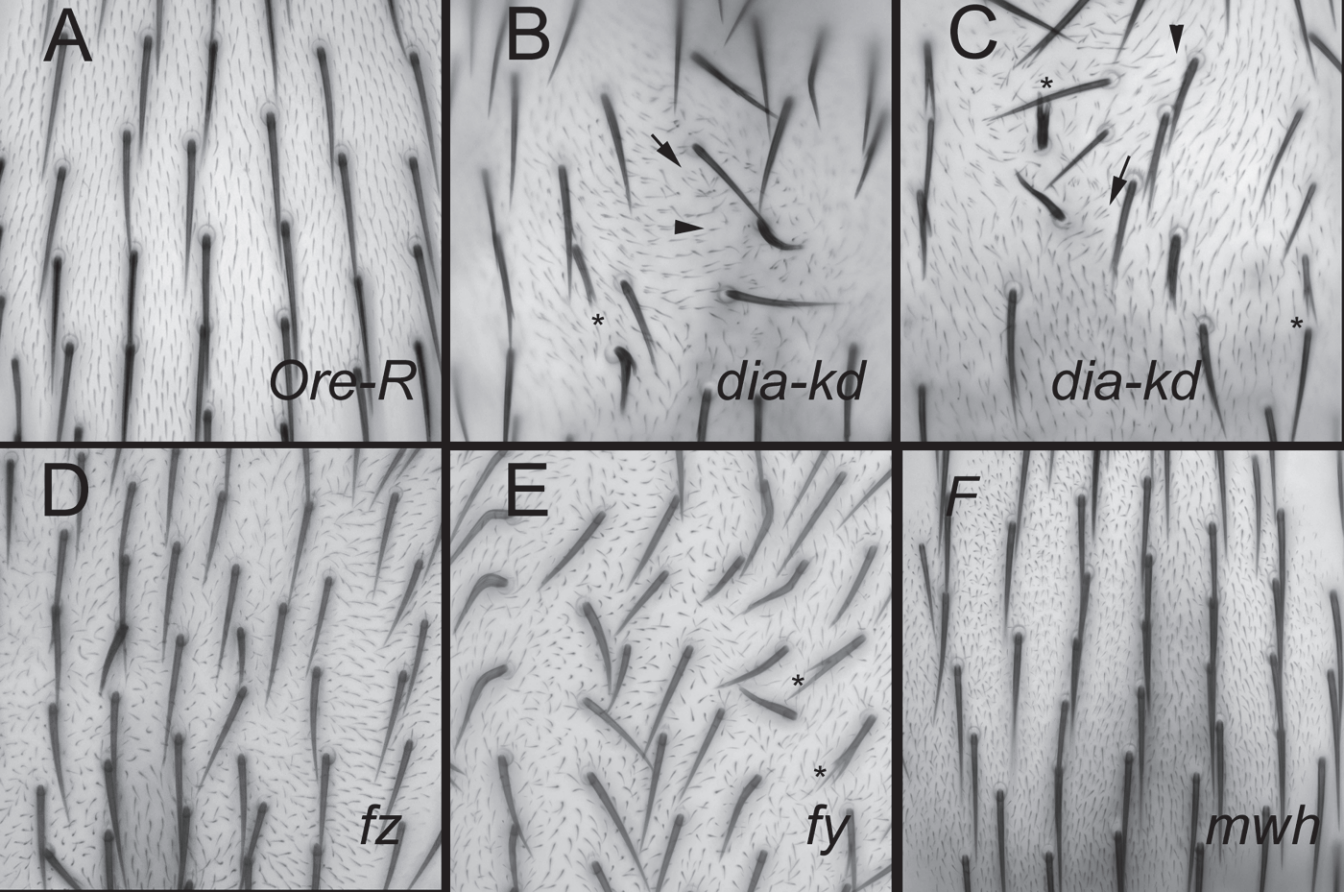
Notum trichome phenotypes. Minimal projections of stacks of bright field images of the notum (dorsal thorax). Note all of the hairs and bristles on the Ore-R (A) point posteriorly (down). In the *dia* knockdowns (B, C) there are polyploid cells (arrows), deformed bristles (asterisks) and many hairs (trichomes) do not point posteriorly. Some of these appear to be diploid (arrowheads). The other panels are of *fz* pathway mutants for comparison. D. *frizzled*, E. *fuzzy*, and F. *mwh*. Note the misoriented and swirling hair polarity in D and E. Note the strong multiple hair cell phenotype in F.

The use of *ap-Gal4* in these experiments leads to minor folds in the wing presumably due to *dia* being knocked down in and leading to cell shape changes in dorsal but not ventral cells. The presence of such distortions makes it difficult to be certain that the abnormal polarity phenotype is a primary defect. To get around the distortion problem we needed to examine a tissue whose shape was not distorted in the experiment. As an alternative to the wing we chose to examine hair polarity on the notum of *ap-Gal4 pTubGal80* ^*ts*^ */+; UAS-dia-RNAi/+* flies (Fig. 2). The overall shape of the notum cuticle is only slightly altered in this genotype and the tissue was not flattened for microscopy. We observed both polyploid mhc (arrow) and diploid mhc (Fig. 2BC). Interestingly, we also routinely observed swirling trichome patterns that are typical of PCP mutants (Fig. 2B–E, arrowheads). Indeed the *dia* kd phenotype on the thorax was similar to the strength seen in null alleles of PCP genes. None of these phenotypes are seen on wild type flies (Fig. 2A). We also examined clones homozygous for the hypomorphic *dia* ^5^ allele [51]. We rarely recovered clones, and when observed in wing discs clones were very small and in adult wings most clone cells were polyploid (Fig. 1H, arrows). The rarity of the clones (typically 0–5 clones per adult wing) precluded a detailed analysis. Polyploid cells were seen both in the wing blade and at the wing margin (S2 Fig.). In addition to polyploid cells we also saw rare multiple hair cells that appeared to be diploid. Further, we saw evidence of cell death due to the generation of clones in the wing. For example, some wings showed evidence of a loss of sections of wing margin (S2 Fig.). Similar phenotypes are seen in a number of mutations that cause cell death [55,56]. We observed bristle phenotypes in these flies, such as a loss of the socket cell and duplicated shafts (S2 Fig.) suggestive of defects in the asymmetric cell divisions that give rise to the bristle sense organ [57,58]. When we examined the notum of flies that contained *dia* ^5^ clones we observed the occasional loss of bristles and small patches of notum with hairs that appeared to be formed by polyploid cells (S3 Fig.). We also saw what appeared to be diploid mhc (S3 Fig.) and potential polarity abnormalities (S3 Fig.). On the whole the *dia* ^5^ clone phenotypes were quite similar to those seen with *dia* knock downs and served to validate the knock downs.

**Fig 3:**
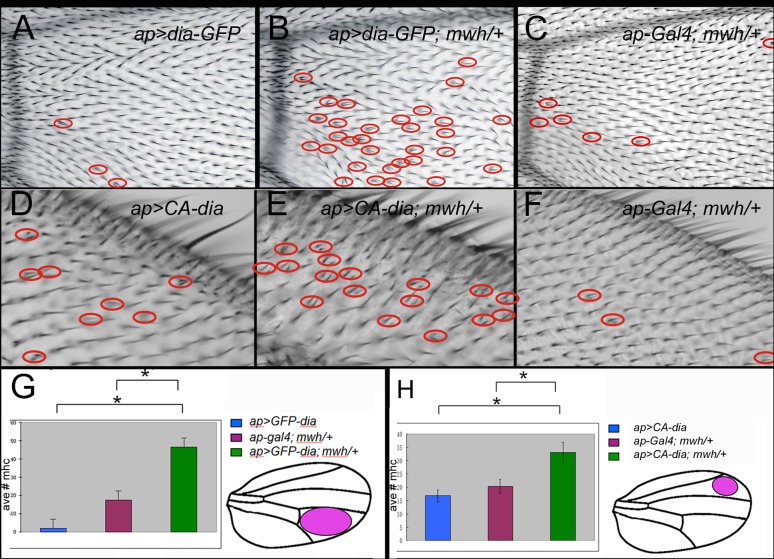
Antagonistic genetic interaction between *dia* and *mwh*. Genotypes are provided on the images. Red circles highlight multiple hair cells. Note the increased number of multiple hair cells in the *ap>dia-GFP; mwh/+* (B) wing compared to *ap>dia-GFP* (A) and *ap-Gal4/+; mwh/+* (C) by themselves. Quantitation is shown in G (p<0.001). Note the increased number of multiple hair cells in the *ap>CA-dia*; *mwh/+* (E) wing compared to *ap>CA-dia* (D) and *ap-Gal4/+; mwh/+* (F) by themselves. Quantitation is in H (p < 0.01). In all of these wings a *ptub-Gal80* ^ts^ transgene was present and a temperature shift was done to restrict the length of time the *dia* transgene was expressed (see text for details). * indicates a significant difference (t-test).

The overexpression of wild type Dia-GFP produced a small number of multiple hair cells (Fig. 3A, red ovals). Since Dia activity is regulated post-translationally it is not surprising that wing cells were only mildly affected by Dia overexpression. To eliminate the ability of cells to post-translationally down regulate the activity of the excess Dia protein we expressed two different constitutively active (CA) Dia variant proteins (DiaΔDad (we refer to this variant as CA-Dia) and FH3FH1FH2 [59]). In both cases they lack the Dad domain which is essential for auto-inhibition. We obtained similar phenotypes from both but primarily used CA-Dia as it was slightly more potent. Consistent with these proteins being potent constitutive activators of actin polymerization we found them to be lethal when expressed by most Gal4 drivers. To get around the lethality we used Gal80^ts^ and temperature shifts to limit CA-Dia expression to a short period of time after cell division had ceased in the wing (1–12 hr starting 24–28 hr awp (either *ap-Gal4 pTubGal80* ^*ts*^ or *ptc-Gal4 pTubGal80* ^*ts*^ were used) [54]. Much stronger phenotypes were seen with *ap-Gal4*, presumably due to the expression of *ptc-Gal4* strongly declining in the 12 hours prior to hair initiation. In wings displaying the strongest phenotypes the wing did not fully expand (Fig. 1B), wing cells were bulged (Fig. 1B, C) and the wing blade was highly folded (Fig. 1B). Many of the hairs pointed in abnormal directions and were short and thick with a collection of very thin distal extensions (that were often branched) (Fig. 1C, large arrows). Many hairs were curved and/or branched, and some cells formed multiple independent hairs (Fig. 1C, small arrows) some of which were very small (Fig. 1C, arrowhead). Such wings are so abnormal it is difficult to interpret the phenotypes. More informative were wings that displayed somewhat weaker phenotypes (Fig. 1D) due to a more limited time of CA-Dia expression. These wings contained cells that formed multiple independent hairs (small arrows), very short hairs (arrowheads), hairs with thickened proximal regions and multiple thin extensions (large arrows), curved hairs, and split hairs (grey arrows). It was common in such wings to find regions where hair polarity was abnormal (Fig. 1D).

**Fig 4:**
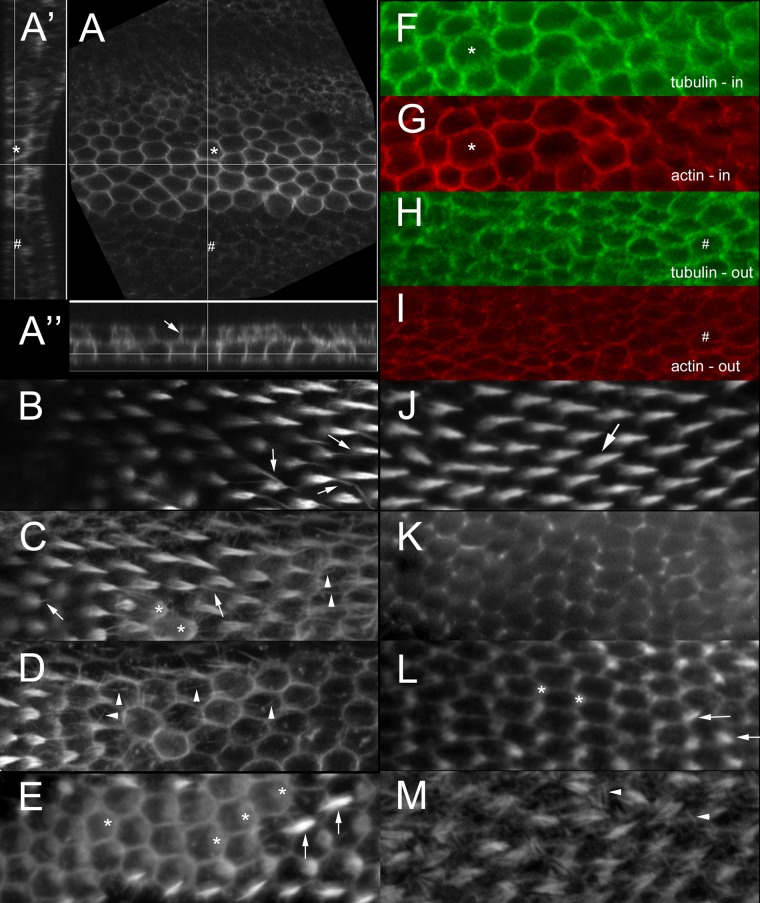
Effect of expression of CA-dia on pupal wings. All panels with the exception of M (*mwh*) are of *ptc>CA-dia* pupal wings. A phalloidin stained pupal wing (A, A’, A”) prior to hair initiation shows increased F-actin staining along the lateral cell membranes (arrows). Both a maximal projection (A) and orthogonal views (A’, A”) are shown. The cells in the middle of the *ptc* domain have increased apical size (*) and are shorter (A’) compared to cells outside the domain (#). B-D. Are each maximal projections of two optical sections separated by 0.3 um from a *ptc>CA-dia* pupal wing stained for F-actin during hair outgrowth. B-D are successive maximal projections of two optical sections as one moves from the dorsal surface ventrally. The wing was not completely flat or orthogonal to the microscope axis. B. Arrows point to hairs that contain a long thin filopodial like distal extension. C. Arrows point to hairs that contain a long thin distal extension. Asterisks mark cells where F-actin accumulation was seen over a larger region of the apical cytoplasm than normal. Arrowheads point to actin filaments/bundles not normally seen in such cells. D. Arrowheads point to actin filaments/bundles not normally seen in such cells. E. A single optical section of a different *ptc>CA-dia* pupal wing stained for F-actin during hair outgrowth. Note the two types of cells. Some have relatively well formed hairs (arrows). Others show delayed hair outgrowth and an unusually large region stained by F-actin (asterisks). F-I are images from a *ptc>CA-dia* pupal wing prior to hair initiation stained for both tubulin (red—G, I) and actin (green—F, H). The upper panels are from inside the *ptc* domain and the lower panels from the same wing but outside of the *ptc* domain. Note the brighter staining and increased lateral accumulation of both F-actin and tubulin in cells inside the *ptc* domain (asterisks) compared to outside of the *ptc* domain (pound sign). J and K are from the same pupal wing as BCD, but in this case from outside of the *ptc* domain so these cells should be phenotypically wild type. The hairs (J—arrow) appear normal. The distinctive ectopic actin filaments/bundles seen in C and D (arrowheads) are not visible in either J or K (K is from just below the apical surface). L is from a region outside of the *ptc* domain from the same wing as E. It shows a region of the wing where hair initiation is occurring (two small hairs are marked by arrows). Note how much smaller the region of apical cytoplasm that stains brightly for F-actin (asterisks) is compared to when CA-Dia is expressed (E). M is a *mwh* mutant wing stained for F-actin. Extra actin filaments are seen in the apical cytoplasm (arrowheads).

We examined pupal wings to directly assess the consequences of expressing CA-Dia before and during the time of hair initiation as well as later in hair morphogenesis (Fig. 4). Once again we limited the period of expression by using *Gal80* ^*ts*^ and a temperature shift. We primarily utilized *ptc-Ga4* as the driver because the animals were healthier, their developmental timing was more reproducible and the weaker phenotypes were easier to interpret. Prior to hair initiation affected cells showed much stronger cortical F-actin staining than is seen wild type cells (Fig. 4A, A’, A”, asterisk vs pound). This cortical staining extended from the apical to basal level of the wing cells (Fig. 4 A”, arrow). The expression of CA-Dia resulted in cells being shorter on the apical/basal axis but having a larger apical surface. At hair initiation two types of cells were seen in the *ptc* domain. Many showed at most minor abnormalities (Fig. 4E, arrows). In others F-actin accumulated over a wider region (Fig. 4CE, asterisks) than normal (Fig. 4L, asterisks) and hair outgrowth was delayed (Fig. 4E, asterisks). In wild type cells F-actin accumulated over approximately 8.7% (0.02sd) of the apical cell surface at hair initiation (Fig. 4L, asterisks), while in the strongly affected *ptc>CA-dia* cells (Fig. 4E, asterisks) this was increased to 33% (0.07sd). This 3.8 fold difference was highly significant (t-test, p = 4 X10^-12^). This suggested that the multiple hair phenotype of CA-Dia is due to cells failing to restrict the location for actin polymerization to a small region around the distal vertex. We also noticed F-actin bundles/filaments in the apical regions of cells apart from the region of hair morphogenesis (Fig. 4CD, arrowheads). These are not seen in wild type (Fig. 4KL) but are seen in *mwh* mutants (Fig. 4M, arrowheads) [37]. In addition we observed thin filopodia like processes that contained actin extending from hairs (Fig. 4BC, arrows). These are not seen in equivalently treated normal wings (e.g Fig. 4J, arrow). These could be related to the thin extensions of hairs seen in the SEM images of *apGal4 pTubGal80* ^*ts*^ */+; UAS-CA-dia/+* wings (e.g Fig. 1C). We also examined CA-Dia expressing pupal wings after hair extension was essentially complete (after 40–42 hrs awp). At this time the wing cells start to flatten and chitin deposition is first detected [60]. The flattened wing cells have a larger apical cell surface and this leads to an increase in the surface area of the wing resulting in the wing folding in the pupal cuticle wing sac. Coincident with the start of chitin deposition actin foci accumulate under the apical surface of the wing [60]. In *ap-Gal4>CA-dia* pupal wings these foci were almost completely eliminated from the dorsal cells but not ventral cells (S4 Fig.) and wing cell flattening was reduced. It seems likely that CA-Dia interferes with chitin and cuticle deposition, which contributes to the CA-Dia wing blade phenotype seen in the SEM (Fig. 1BC).

Dia is known to also regulate microtubules [46]. We examined the distribution of microtubules in wing cells that expressed CA-dia and found increased cortical tubulin immunostaining that was similar to the F-actin staining seen in those cells (Fig. 4FG vs HI). In general, the tubulin abnormalities appeared less severe than the actin abnormalities but it remains an open question as to which is more important in producing the mutant phenotypes.

### *dia* and bristle morphogenesis

The sensory bristles of Drosophila are another convenient cell type to examine the role of the actin and microtubule cytoskeletons in polarized growth [34,61,62]. In experiments done to examine the wing phenotype (see above) we observed that the expression of CA-Dia resulted in a range of highly abnormal bristles. These included split bristles, bent bristle and dramatically shortened bristles (S3 Fig.). The severity of the phenotype was dependent on the length and timing of *CA-dia* expression and we did not examine this quantitatively. In principle, bristle abnormalities can arise from defects in bristle growth [34,61,62] or due to the collapse of extended bristles due to defects in cuticle deposition [63]. Indeed, collapse can give rise to very short bristles similar to those obtained after expression of CA-Dia (S3 Fig.) [63]. We carried out limited in vivo imaging experiments on bristles where CA-Dia was expressed and observed typical *CA-dia* phenotypes during bristle outgrowth (S3 Fig.). Thus, at least part of the bristle phenotype is due to defects in primary bristle outgrowth. We cannot rule out a contribution due to a failure to maintain bristle morphology, although we did not detect any evidence for this mechanism. We also observed abnormal aristae in *ap>CA-dia* flies (S5 Fig.). The abnormalities included a short and thick central core (arrows) and short, split and wispy side branches (arrowheads). We previously obtained the former after injection of microtubule inhibitors into pupae and the latter after injection of actin antagonists [64].

We detected more modest phenotypes when *dia* was knocked down in bristle forming cells. The most common phenotypes were bent and/or split bristles and wispy bristle tips (S3 Fig.) and these were not found on all animals.

### *mwh* and *dia* interact genetically

The experiments described above established that too much Dia activity in pupal wing cells resulted in a number of dramatic wing and hair morphology abnormalities including several that were PCP like phenotypes. This included many cells that formed multiple hairs, some of which were very short and hairs with abnormal polarity. Further, many of the cells that formed multiple hairs formed 3 or more hairs. The only PCP gene that shares this set of phenotypes is *mwh* [3,36]. This suggested that the *mwh* mutant phenotype could be due at least in part to excess Dia activity. We carried out a number of genetic experiments to test this hypothesis. When *GFP-diaphanous* was over expressed (*UAS-GFP-dia/ap-Gal4 ptub-Gal80* ^*ts*^ at 29°C), only occasional double hair cells were observed (Fig. 3A). A reduction in the dose of *mwh* (*UAS-GFP-dia/ ap-Gal4 ptub-Gal80* ^*ts*^ *; mwh/+*) resulted in a large increase in the number of cells producing two hairs (Fig. 3B). We quantified this phenotype in the region distal to the posterior cross vein (Fig. 3G) and found the average number of double-hair cells was significantly increased (P<0.001, t-test).

We also found similar interactions in wing cells that expressed constitutively active Dia (*UAS-GFP-CA-dia/ ap-Gal4 ptub-Gal80* ^*ts*^ vs *UAS-GFP-CA-dia/ ap-Gal4 ptub-Gal80* ^*ts*^; *mwh/+*) (Fig. 3D–F). Once again the difference in the number of multiple hair cells was significant (Fig. 3H) (P<0.01, t-test). These data established that there is an antagonistic relationship between *mwh* and *dia*.

### Dia and Mwh co-localize in wing cells and growing hairs

**Fig 5:**
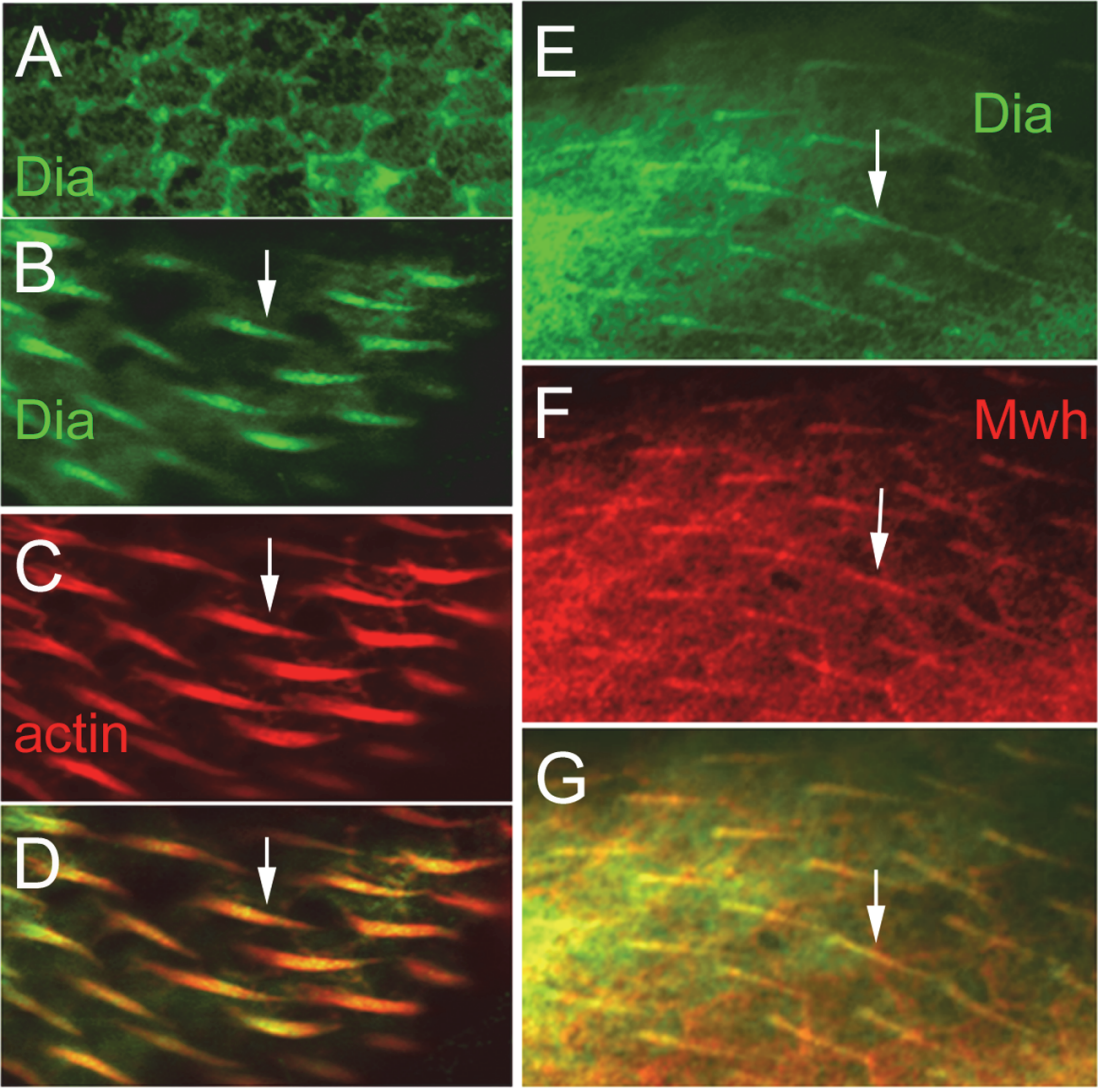
Localization of Dia and Mwh in pupal wings. In *ptc>dia-GFP* pupal wings prior to hair formation Dia localizes at the cell periphery (A). During hair elongation (B-G) Dia preferentially localizes in a punctate manner to the hair. At 34 hr awp (after white prepupae) both actin and Dia concentrate in the growing hairs (B-D, arrows). Later in wing development (E-G)(~40 hrs awp) the hair has moved to the center of the cell and Dia and Mwh are preferentially localized to the hair (arrows). Thus, the two proteins are localized in the same region of wing cells and are in position to interact.

To determine where Dia was localized in pupal wing cells we immunostained pupal wings expressing GFP-Dia. Dia preferentially localized to the cell periphery prior to hair initiation (Fig. 5A) and later was found in growing hairs (Fig. 5B). As was described previously Mwh was preferentially found at the proximal side of wing cells prior to hair initiation and was then found in the growing hairs. We also co-immunostained for these two proteins in pupal wing cells and observed substantial co-localization in growing hairs (Fig. 5EFG). We quantified this and found an average correlation coefficient for immunostaining in the hair of 0.5513 (S6 Fig.). These experiments established that Mwh and Dia are found in the same region of pupal wing cells and could potentially interact directly.

### Mwh and Dia interact physically

**Fig 6:**
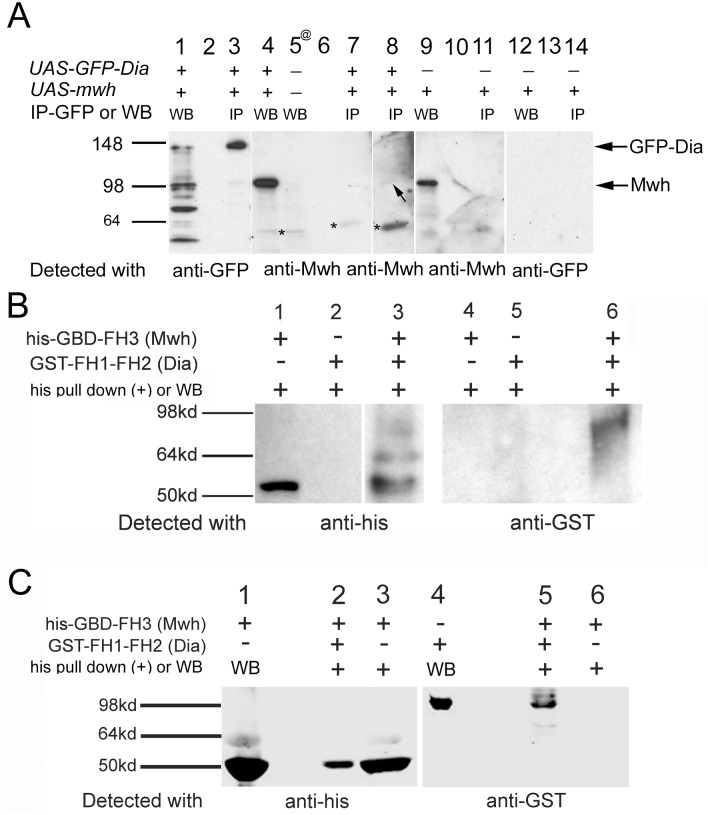
Co-immunoprecipitation experiments show that Dia and Mwh can interact physically. Co-Immunoprecipitation experiment from cells that expressed both full length Dia-GFP and Mwh (A). The arrow points to the weak band that is the size expected for Mwh. The asterisks point to a band recognized by anti-Mwh antibody that is likely due to cross reaction. His pull downs of E. coli extracts made from cells that expressed his-GBD-FH3(Mwh) and GST-CC-FH1-FH2(Dia) pulled down the Dia fragment (B). Note that a protein the expected size of GST-CC-FH1-FH2(Dia) is recovered in a his pull down (lane 6) and this requires the co-expression of Mwh (compare lane 6 and lane 5). This indicates that the his pull down of GST-CC-FH1-FH2 is due to an interaction with Mwh. His pull downs of purified his-GBD-FH3(Mwh) and GST-CC-FH1-FH2(Dia) (C). There is a strong his pull down of GST-CC-FH1-FH2 (lane 5) arguing there is a direct interaction between Mwh and Dia.

The interactions detected in genetic experiments could be a mediated by a direct or indirect interaction between Mwh and Dia. To distinguish between these two models we first attempted to determine if Dia and Mwh could be co-immunoprecipitated from wing disc extracts. When we precipitated protein using an anti-GFP antibody we detected a band the size of GFP-Dia ((Fig. 6A, lane 3) that was only seen when GFP-Dia was expressed (Fig. 6, compare lanes 3 and 14) indicating that the IP was specific. When we probed the precipitated material with anti-Mwh antibody we did not observe a convincing band at the expected size of Mwh (Fig. 6A, lane7). Increasing the exposure resulted in the appearance of a faint band at that size (Fig. 6A, lane 8- arrow). Assuming this band was Mwh we estimated only 0.04% Mwh was associated with Dia. This was suggestive (but not compelling) evidence that these two proteins could be in a common complex. Why might the co-IP be so weak if the two proteins were in a common complex? One possibility is that the proteins could be present in a complex that was not stable to the experimental procedure. Alternatively, both proteins are likely activated by the binding of Rho1-GTP and it is possible that only a small fraction of one or both proteins is in an active form. Inactive proteins might not be able to interact. Another possibility is that at least one of these proteins (e.g. Dia) is preferentially found in one or more alternative complexes that do not contain Mwh. Only “left over” Dia might be present in a complex with Mwh. Finally, we used wing discs and not pupal wings (which are much more difficult to dissect without fixation) as the source of material and this difference in developmental stage could contribute to either of the latter two possibilities. This seems quite plausible as *mwh* expression increases sharply around the time of hair intiation [47].

To obtain more definitive evidence as to whether or not these two proteins could interact physically we chose to carryout in vitro experiments. To eliminate possible problems due to the proteins being auto-inhibited we examined protein fragments where known (Dia) and putative (Mwh) auto-inhibition sites were removed. The use of a fragment of Mwh was also necessary as we were unable to purify full length Mwh in a soluble form. We tested a His tagged Mwh-GBD-FH3 fragment and a GST tagged Dia-CC-FH1-FH2 fragment. In a His pull down assay on E.coli extracts expressing the two proteins we found the GST tagged CC-FH1-FH2 fragment of Dia was pulled down (Fig. 6B, lane 6). In the absence of his-Mwh-GBD-FH3 (Fig. 6B, lane 5) there was no pull down of GST-Dia-CC-FH1-FH2 indicating that the pull down was specific for a Mwh-Dia interaction. The E. coli extracts were not expressing any other fly proteins so it seemed quite likely that this interaction was a direct one. To confirm this was the case the protein fragments were purified (S7 Fig.) and tested in a his pull down. The purified proteins gave a strong co-IP (Fig. 6C, lane 5) confirming that these two protein fragments can interact directly. These results suggest that the antagonistic genetic relationship between *dia* and *mwh* is due to a direct physical interaction. We note that the GST-Dia-CC-FH1-FH2 fragment did not contain the DD so that domain is not responsible for the interaction. The structural basis for the interaction is unknown at this point and determining that will require additional experimentation.

## Discussion

### The function of Dia in the morphogenesis of wing hairs

Growing wing hairs contain actin filaments and a variety of data establishes that these filaments are important for hair morphogenesis [16,17,18,19,22–25,27,28]. It is likely that long actin filaments are important and this led us to suspect that one or more of the 6 Drosophila formins would be important for hair morphogenesis. In knock down experiments only *dia* produced a strong hair phenotype. We cannot rule out any of the other formins also functioning in hair morphogenesis as we did not determine the effectiveness of the knock downs and we did not do experiments to rule out redundancy. Indeed the very weak phenotype seen with a knock down of *CG32138* could be due to either of these. For the *DAAM* formin published standard genetic tests argue against a role in wing hair morphogenesis [65].

A range of phenotypes were seen with both loss (lof) and gain of function (gof) conditions for *dia* that implicate Dia in both hair morphogenesis and PCP. Many of the hair morphology phenotypes (e.g. branched hairs, thickened hairs) seem likely to be related to the abnormal activation of the actin cytoskeleton. Given the extensive literature on the role of Dia in regulating the actin cytoskeleton these phenotypes were not surprising. The involvement in PCP was less expected. The severity of the hair morphology phenotypes complicated but did not eliminate our ability to examine the role of Dia in PCP. Alterations in Dia activity led to the formation of multiple hair cells, hairs of abnormal polarity and swirling hair patterns, all of which are classic phenotypes of PCP mutants [1,2,3].

### *dia* and *mwh* in wing PCP

The *fz* PCP pathway normally functions to restrict the activation of the cytoskeleton to a small region around the distal most part of wing cells [3]. When CA-Dia was expressed actin polymerization was activated over a larger cellular region than normal and this resulted in multiple hair cells. Thus, CA-Dia interfered with the ability of the PCP pathway to restrict activation of the cytoskeleton to a small part of the cell. This suggested that spatially limiting Dia activation could be a downstream function of the PCP pathway. The *mwh* gene is the most downstream known component of the pathway and hence is a candidate to mediate such a function. Our observations are consistent with this hypothesis. Notably, the multiple hair cell phenotype associated with CA-Dia expression included the formation of tiny hairs, which is a distinctive phenotype of *mwh* mutants [3,36,37,66] and no other PCP mutants. In addition, the expression of CA-Dia resulted in the formation of ectopic actin filaments (or bundles of filaments) just below the apical surface of wing cells, which is also observed in *mwh* mutants but not other PCP mutants [37]. Further, we observed antagonistic genetic interactions between Dia and Mwh, we found the two proteins partially co-localize in pupal wing cells and we established that the two proteins could directly interact. We suggest that the binding of Mwh to Dia could prevent Dia from activating the actin cytoskeleton and in this way the PCP pathway could localize the activation of the cytoskeleton. Our data however, cannot rule out the possibility that the antagonistic relationship between *dia* and *mwh* is due to both independently regulating the actin cytoskeleton. This hypothesis needs to be considered given that we did not see a strong co-IP from wing discs.

In our experiments where CA-Dia was expressed the *fz/stan* pathway was functional but it was not able to block the hyper activation of the cytoskeleton. Indeed, the consequences of CA-Dia expression were more severe than loss of *fz/stan* pathway function. This could be due to either the over expression of CA-Dia overwhelming the system or to the constitutively active nature of the protein. Of course both factors could be important. It is worth noting that the *fz/stan* system appears to be finely tuned as both gain and loss of function mutations give rise to PCP phenotypes and it is unable to scale to insure a single distally pointing hair is formed in polyploid cells [53]. Hence it is not surprising that it appears limited in its ability to deal with cytoskeletal perturbations.

In vertebrates the DAAM formin has been implicated in PCP [67] and in mediating an interaction between Dishevelled and Rho. There is a DAAM in Drosophila but it does not appear to play a role in PCP [65], rather it appears to function in trachea development. Additional systems will need to be studied to determine if any other members of the formin family play a role in PCP.

### Hair initiation and *dia*

When we expressed CA-Dia in pupal wing cells we observed increased accumulation of F-actin, consistent with the well-known activity of Dia in other cells types/systems. Prior to hair initiation the increased F-actin was largely restricted to the lateral cell membranes. Thus, the presence of CA-Dia was not sufficient to induce hair formation and other factors must be important in regulating the initiation of hair morphogenesis. The expression of many genes changes dramatically around the time of hair initiation [47] and one or more of these are good candidates for such a function. One of these, *shavenoid* is a particularly strong candidate as mutations in this gene lead to the loss of hair formation in some cells and a substantial delay in initiation in others [68].

When hair initiation began in cells expressing moderate levels of CA-Dia increased F-actin was primarily seen over the distal apical region of the cell. This region was much larger than normal but still only represented a minority of the cell. Thus, even in the presence of CA-Dia the wing cells still retained significant spatial control over actin polymerization. This seems likely to be due to the PCP mediated localization of factors that regulate the actin cytoskeleton as there was still a distal bias to activation of the cytoskeleton. The Mwh protein could serve this role by inhibiting endogenous Dia and/or CA-Dia proximally leading to a higher level of actin cytoskeleton activation distally where both CA-Dia and endogenous Rho1 activated Dia could stimulate actin polymerization. Mwh could also be acting independently of its interaction with Dia. There is reason to believe that wing PCP involves both negative regulation proximally and positive regulation distally [1,37,69]. The Dsh protein is a candidate for being a positively acting factor for mediating this distal bias. Dsh is localized on the distal side of wing cells [70] and in cultured cells it has been shown to regulate Dia activity by the local activation of Rho1 [71]. This was in the context of regulating spindle orientation but it is easy to see how a similar mechanism could lead to the preferential activation of Dia on the distal side of wing cells. The endogenous Dia protein being preferentially activated on the distal side of wing cells could bias the location for hair initiation even in the presence of uniformly distributed CA-Dia.

### *dia* gain and loss of function mutations both give rise to multiple hair cells

It is notable that both *dia* lof and gof genotypes lead to multiple hair cells. It is an unusual property shared by most of the genes of the *fz* pathway that both gof and lof genotypes lead to similar mutant phenotypes [72,73]. In these cases it is thought that asymmetric protein accumulation is essential for function and this is lost both when an active protein is not present or when it is over expressed and over saturates the asymmetry system. We also know that mutations that decrease the activity of the actin cytoskeleton [20,24] and treatment with actin cytoskeleton inhibitors [27] give rise to multiple hair cells. Here it is thought that the multiple hairs result from the lower activity of the cytoskeleton leading to a failure to properly refine the area when the cytoskeleton is activated to insure only a single hair is formed [25,73]. This can explain the *dia* lof mhc phenotype. The hyper-activation due to the over expression of CA-Dia leads to the activation being spread over a much larger region and this also appears to lead to multiple hairs forming. Proper PCP in the fly wing appears to require the function of an efficient and highly regulated cytoskeleton.

### Dia and bristle morphogenesis

Diaphanous has primarily been studied with respect to its regulation of the actin cytoskeleton but it is also known to regulate the microtubule cytoskeleton [46]. Indeed we detected changes in both cytoskeletons associated with the expression of CA-Dia. The expression of CA-Dia led to particularly dramatic alterations in bristle morphogenesis. The phenotypes ranged from branched, bent and shortened bristles to “stubs” where the bristle was less than 10 percent of its normal length. The branched and bent bristles were reminiscent of those induced by the injection of inhibitors of actin polymerization such as cytochalasin D and latrunculin A [62] and we suspect they are due to effects of CA-Dia on the actin cytoskeleton. We are aware of two previous reports of a “stub” bristle phenotype. Such bristles were seen after the injection of microtubule antagonists such as colchicine into pupae [62]. Stub bristles were also found to arise from the collapse of long bristles due to a failure to form a functional cuticle [63]. In vivo imaging of pupae that expressed CA-Dia in growing bristles showed that severe bristle abnormalities were present during outgrowth. We take these observations as support for the hypothesis that the stub bristle phenotype was due to the effects of CA-Dia on the microtubule cytoskeleton. The fat short central segment of the arista seen in *ap>CA-dia* flies is also likely to be due to effects on the microtubule cytoskeleton [64]. That Dia regulates both the actin and microtubule cytoskeletons complicates the interpretation of mutant phenotypes. We have not seen microtubule abnormalities in *mwh* mutant cells so we think it likely that the *mwh-*like hair phenotypes are due to effects of CA-Dia on the actin cytoskeleton but specific Dia effector mutations will be required for an unambiguous answer.

## Methods and Materials

### Fly Genetics

All flies were raised at 25°C unless otherwise stated. Unless otherwise stated stocks were obtained from the Bloomington *Drosophila* stock center at the Indiana University or were generated in our lab and previously described [36]. *UAS-diaΔDad* (aka *CA-dia*) and *UAS-FH3FH1FH2* transgenic flies were generous gifts from Mark Peifer at the University of North Carolina. To direct transgene expression, we used the Gal4/UAS system [74]. For immunostaining, white pupae were collected and grown at 25°C or 29.5°C for varying lengths of time before fixation. For Western blotting and co-immunoprecipitation, the wing discs were dissected from third instar larva of flies grown at 25°C. The Genbank protein number for Mwh is AAF478454 and Dia is AAF53922.

### Imaging

**Adult wings**. Adult wings were dehydrated in 100% ethanol and then mounted in Euparal. To visualize hairs on the notum thoraxes were mounted in Gary’s magic Mountant. Spacers were used to keep the cover slip from crushing the tissue. Mounted samples were examined under the bright field microscope. Images were obtained using a Spot-RT digital camera (Diagnostics Instruments) on a Zeiss Axioskop II microscope. In some cases Metamorph was used to obtain 3D stacks of images. Minimum projections of such stacks were used to increase the effective depth of field and to allow us to visualize 3D structures such as the hairs on the notum.

**Quantitative analysis of the genetic interaction between *dia* and *mwh* and between *CA-dia* and *mwh*.** To characterize the genetic interaction between *dia* and *mwh*, we scored the number of multiple hair cells in the region distal to the posterior cross vein in 20 wings. The significance of the difference was evaluated using two-sample unequal variance Student t-Test. A similar method was used to quantify genetic interaction between *CA-dia* and *mwh*.

**Image Acquisition and Analysis**. We used ImageJ to quantify aspects of both confocal images of pupal wings and bright field images of adult wings. Pupal wings were examined by confocal microscopy using a Zeiss 510 Meta confocal microscope.

As evidence for the co-localization of Dia and Mwh in the hair, the grey scale values of the immunostaining signal for GFP-Dia and Mwh were individually determined along a line drawn across the proximal region of 5 different hairs (an example is shown in S6 Fig.). The purpose for the line not crossing the whole length of the hair is to avoid crossing the cell periphery where Mwh had strong staining. The correlation coefficient between plot values of GFP-Dia and Mwh staining were calculated using Microsoft Excel. The average correlation coefficient was calculated based on values from 5 different hairs.

**Immunostaining**. Immunostaining was done on paraformaldehyde fixed samples by standard procedures as described previously [36].

Polyclonal rabbit anti-GFP antibodies and monoclonal mouse anti-GFP antibodies were purchased from Invitrogen. A rat polyclonal anti-Mwh antibody was generated in our lab [36]. Alexa 568 phalloidin was purchased from Molecular Probes. Alexa 488- and Alexa 568-conjugated secondary antibodies were purchased from Molecular Probes.

### Molecular Biology

**Plasmid Constructs**. The *UAS-mwh-GBD-FH3–3HA* construct was made by amplifying the appropriate segment of *mwh* encoding the GBD-FH3 domain adding Gateway cassette sequences (Invitrogen) on both sides. The primers are 5′-GGGGACAAGTTTGTACAAAAAAGCAGGCTTCCAAAAC

ATGTACAGCAAGGAAAACCAGCG-3′ and 5′-GGGGACCACTTTGTACAAGAAA

GCTGGGTCGATGCCCTCGTCCTCGTG-3′. The cDNA was cloned into Donor 221 vector (Invitrogen) to generate entry clone which was then cloned into pTWH, one of the Carnegie Gateway Vectors constructed by T. Murphy to generate expression constructs with C-terminal 3HA tag.

To make *UAS-GBD-FH3-GFP* construct for examining over expression phenotype, the entry clone of GBD-FH3 described above was cloned into pTWG, one of the Carnegie Gateway Vectors constructed by T. Murphy to generate an expression construct with a C-terminal GFP tag.

*UAS-GFP-dia* and *UAS*-*CA-dia* constructs were generated in Mark Peifer lab at the University of North Carolina.

**Purified proteins**. The 6His-GBD-FH3(Mwh) protein was produced by subcloning DNA encoding aa 60–491 into pET28a and GST-CC-FH1-FH2(Dia) protein was produced by subcloning the region encoding aa 448–1029 into pGEX. They were expressed in E. coli and purified by standard techniques such as affinity chromatography and molecular size exclusion chromatography.

**Co-immunoprecipitation and Western blotting**. 300 wing discs were dissected from third instar larvae for immunoprecipitation assays and an additional 15 wing discs as extract for use as a positive control for Western blotting (WB). Standard immunoprecipitation and WB procedures were performed at 4°C [36]. SeeBlue Plus2 Pre-Stained Protein Standard (Invitrogen) was used as protein size marker and 3–8% premade NuPAGE Tris-Acetate gels (Invitrogen) was used to separate proteins.

Polyclonal rabbit anti-GFP antibodies were used for co-immunoprecipitation of GFP-Dia and Mwh. Mouse anti-GFP antibody and rat anti-Mwh antibodies were used in WB to detect GFP-Dia and Mwh respectively. Detection was by chemiluminescence (Amersham) in early experiments and later by infrared fluorescence (Licor)

## Supporting Information

S1 Fig Arp2/3 complex proteins function in hair morphogenesis. Transgene mediated RNAi was driven by *ptc-Gal4*. *Aprc3a* (A) and *Arpc3b* (BC) knockdowns are shown as examples. The black arrows point to double hair cells, black arrowheads to swollen bases, red arrows to hairs that have fallen over and are not well aligned with their neighbors, red arrowheads to split hairs and the asterisk to a region where a it appears that a cell did not form a hair.
(TIF)

S2 Fig Wing phenotypes seen with *dia* ^5^ clones. Polyploid cells at the margin produce oversized (A, B) hairs and bristles and in some cases duplicated bristles that lack socket cells. Evidence of cell death is seen by the formation of wing nicks (C).
(TIF)

S3 Fig Bristle phenotypes associated with altered *dia* activity. A variety of bristle abnormities are seen in part of a tergite (dorsal abdomen) in *neur-Gal4 ptub-Gal80* ^ts^/*UAS-CA-dia* flies. The arrows point to bristles that show the stub phenotype. A pair of abnormal scutellar bristles (arrows) from a *neur-Gal4 ptub-Gal80* ^ts^/*dia-RNAi* fly. The adult notum of a *neur>CA-dia*, *actin-GFP* fly used for in vivo imaging (C). The arrow points to a severely abnormal bristle that was followed in time lapse. The insert shows an image from the in vivo imaging experiment where the relevant bristle was abnormal during bristle growth. A notum with a putative *dia* ^5^ clone (D) is missing a bristle, contains polyploid cells (arrow) and diploid hair cells (arrowhead). Also note the abnormal hair polarity. Highly abnormal bristles from *ap>CA-dia; ptub-Gal80* ^*ts*^ */+* notums (arrows) (E, F). The severly affected bristles are macrochaetae and the nearby relatively normal bristles are microchaetae.
(TIF)

S4 Fig CA-dia expression leads to defective hairs and a loss of actin foci associated with chitin deposition. Starting at the dorsal surface and moving ventrally images of *ap-Gal4 pTub-Gal80* ^*ts*^ */+; UAS-CA-dia/+* pupal wings stained for F-actin. Each image is a maximum projection of 3 optical sections that represent 0.6um along the dorsal/ventral axis. Arrows in A and B point to multiple hairs cells. The arrows in C point to ectopic actin filaments not normally seen in wing cells. The arrows in H point to actin foci in ventral cells found associated with chitin deposition. These are missing from the dorsal cells. The arrows in K point to normal ventral hairs.
(TIF)

S5 Fig The phenotype of CA-Dia in the arista. Oregon R (A) and *ap>CA-dia* (B) arista are shown. The arrow points to the central core which is grossly short and fat in *ap>CA-dia*. The arrowheads point to laterals which are short and deformed in *ap>CA-dia*.
(TIF)

S6 Fig Dia and Mwh co-localize in growing hairs. The relative intensity of immunostaining for Dia-GFP (green) and Mwh (red) along the proximal part of a growing hair is shown. The presence of substantial Mwh at the cell periphery prevented us from extending the line of measurement to the full length of the hair.
(TIF)

S7 Fig Evidence that the his^6^-GBD-FH3(Mwh) and GST-CC-FH1-FH2 proteins used were pure. The purified proteins were examined by PAGE and western blotting to confirm their purity. The GST-CC-FH1-FH2 protein migrates slightly faster than the 94 kd marker and the his-GBD-FH3 protein migrates at approximately 45kd.
(TIF)
