# Supplementary figures and images for: The diaphanous Gene of Drosophila Interacts Antagonistically with multiple wing hairs and Plays a Key Role in Wing Hair Morphogenesis
Source: PLoS One. 2015 Mar 2;10(3):e0115623. doi: 10.1371/journal.pone.0115623 (PMC4346269; doi:10.1371/journal.pone.0115623)

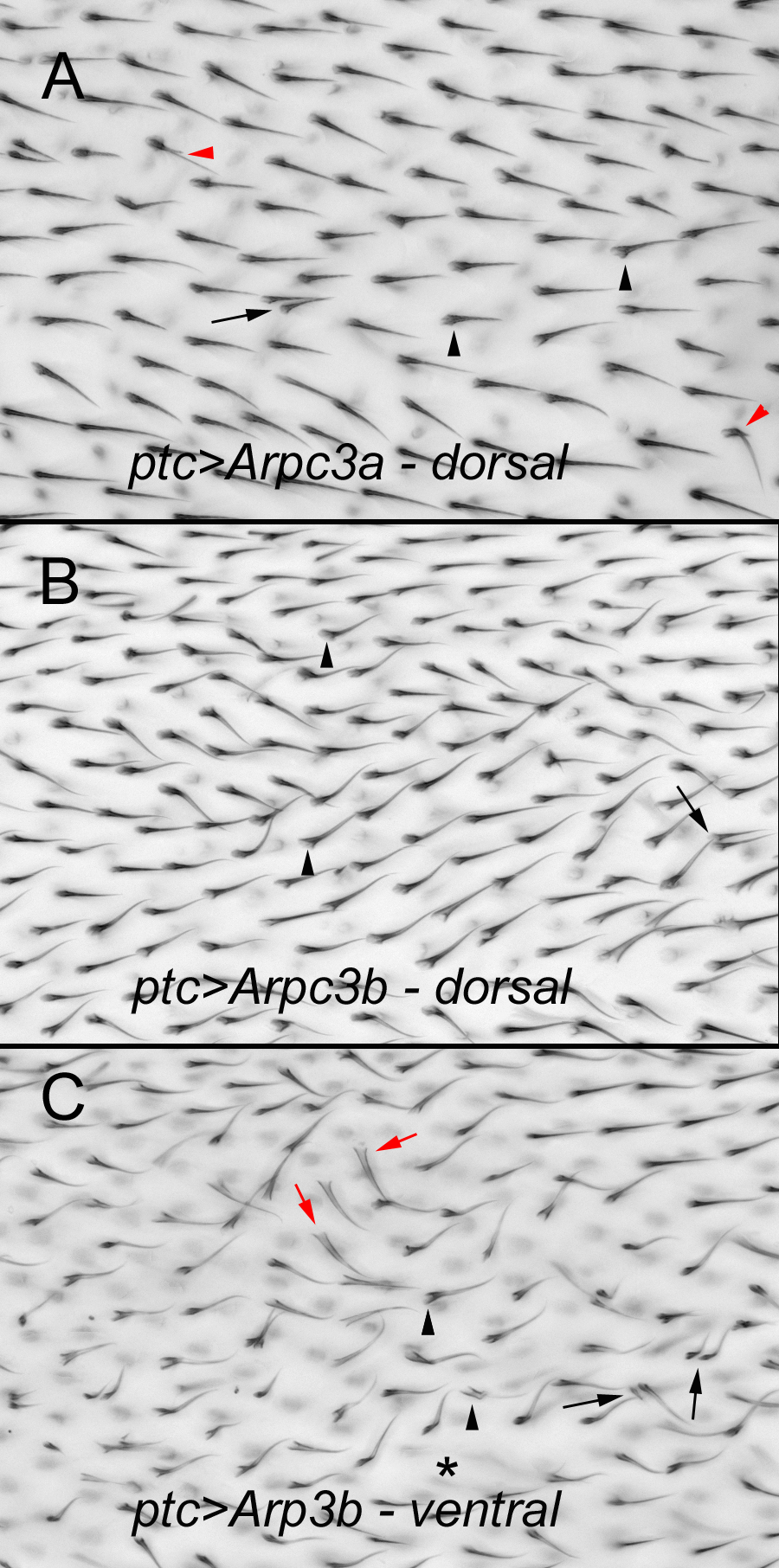

Supplement: S1 Fig — Transgene mediated RNAi was driven by ptc-Gal4. Aprc3a (A) and Arpc3b (BC) knockdowns are shown as examples. The black arrows point to double hair cells, black arrowheads to swollen bases, red arrows to hairs that have fallen over and are not well aligned with their neighbors, red arrowheads to split hairs and the asterisk to a region where a it appears that a cell did not form a hair. (TIF) [file pone.0115623.s001.tif]

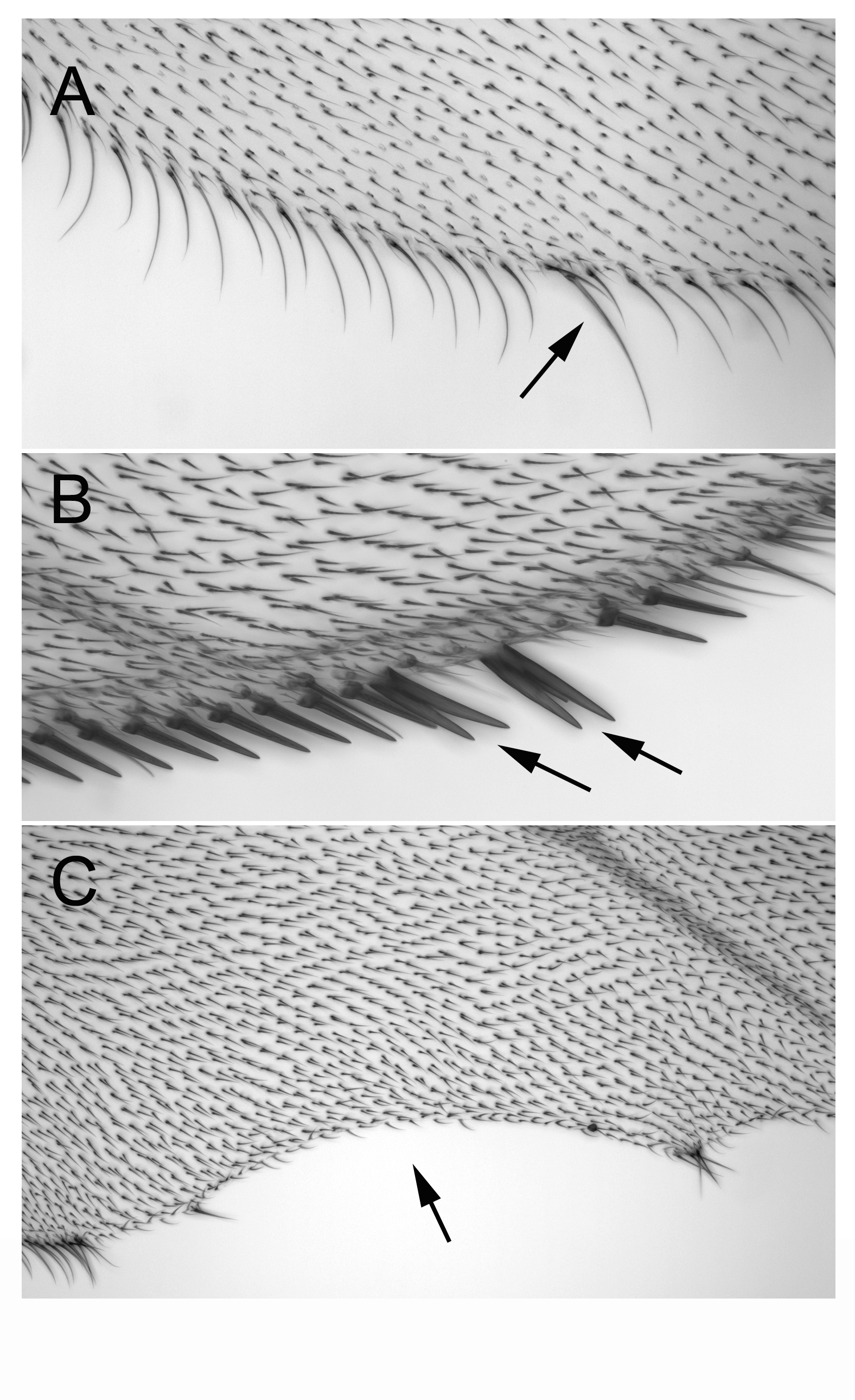

Supplement: S2 Fig — Polyploid cells at the margin produce oversized (A, B) hairs and bristles and in some cases duplicated bristles that lack socket cells. Evidence of cell death is seen by the formation of wing nicks (C). (TIF) [file pone.0115623.s002.tif]

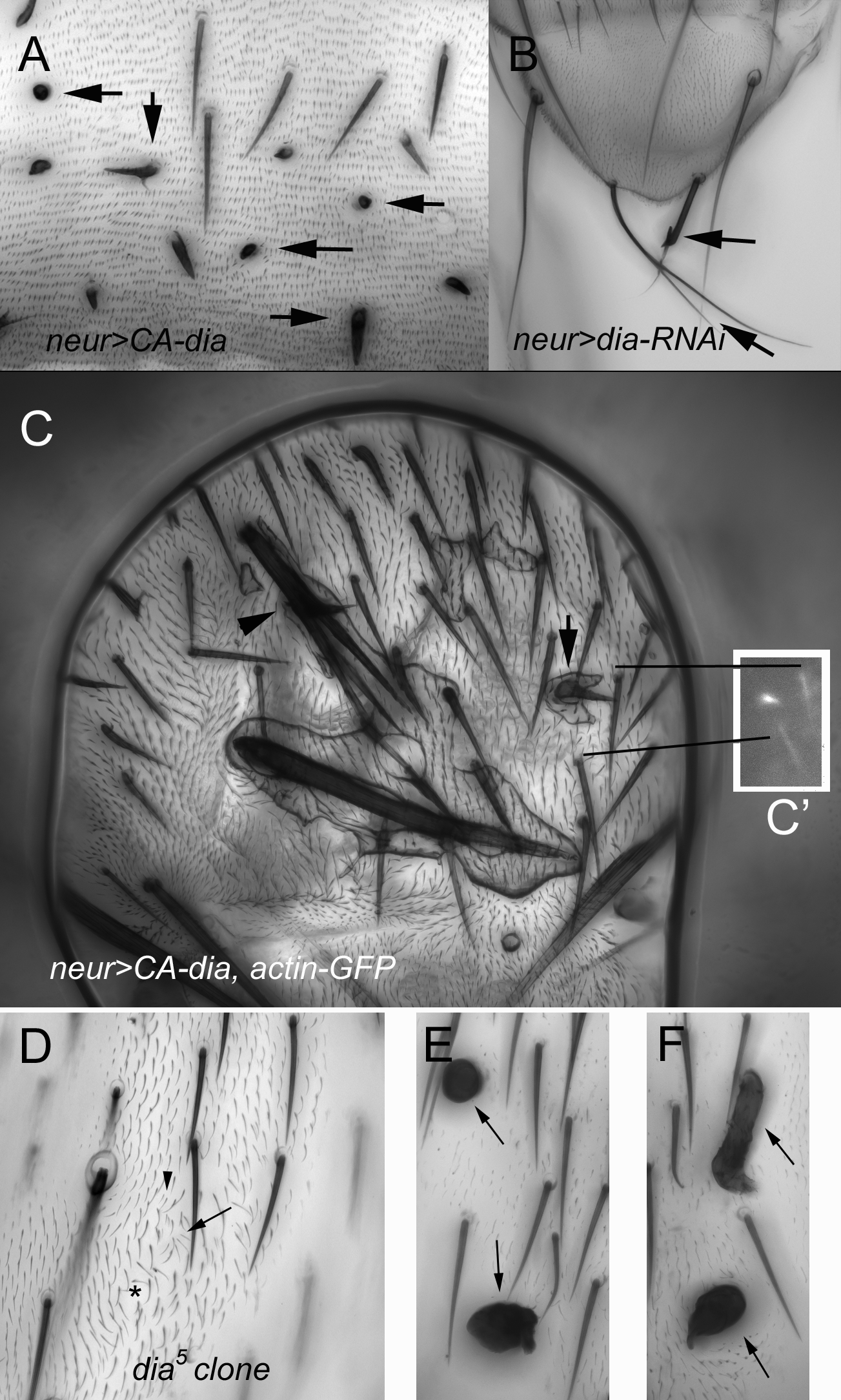

Supplement: S3 Fig — A variety of bristle abnormities are seen in part of a tergite (dorsal abdomen) in neur-Gal4 ptub-Gal80 ts/UAS-CA-dia flies. The arrows point to bristles that show the stub phenotype. A pair of abnormal scutellar bristles (arrows) from a neur-Gal4 ptub-Gal80 ts/dia-RNAi fly. The adult notum of a neur>CA-dia, actin-GFP fly used for in vivo imaging (C). The arrow points to a severely abnormal bristle that was followed in time lapse. The insert shows an image from the in vivo imaging experiment where the relevant bristle was abnormal during bristle growth. A notum with a putative dia 5 clone (D) is missing a bristle, contains polyploid cells (arrow) and diploid hair cells (arrowhead). Also note the abnormal hair polarity. Highly abnormal bristles from ap>CA-dia; ptub-Gal80 ts /+ notums (arrows) (E, F). The severly affected bristles are macrochaetae and the nearby relatively normal bristles are microchaetae. (TIF) [file pone.0115623.s003.tif]

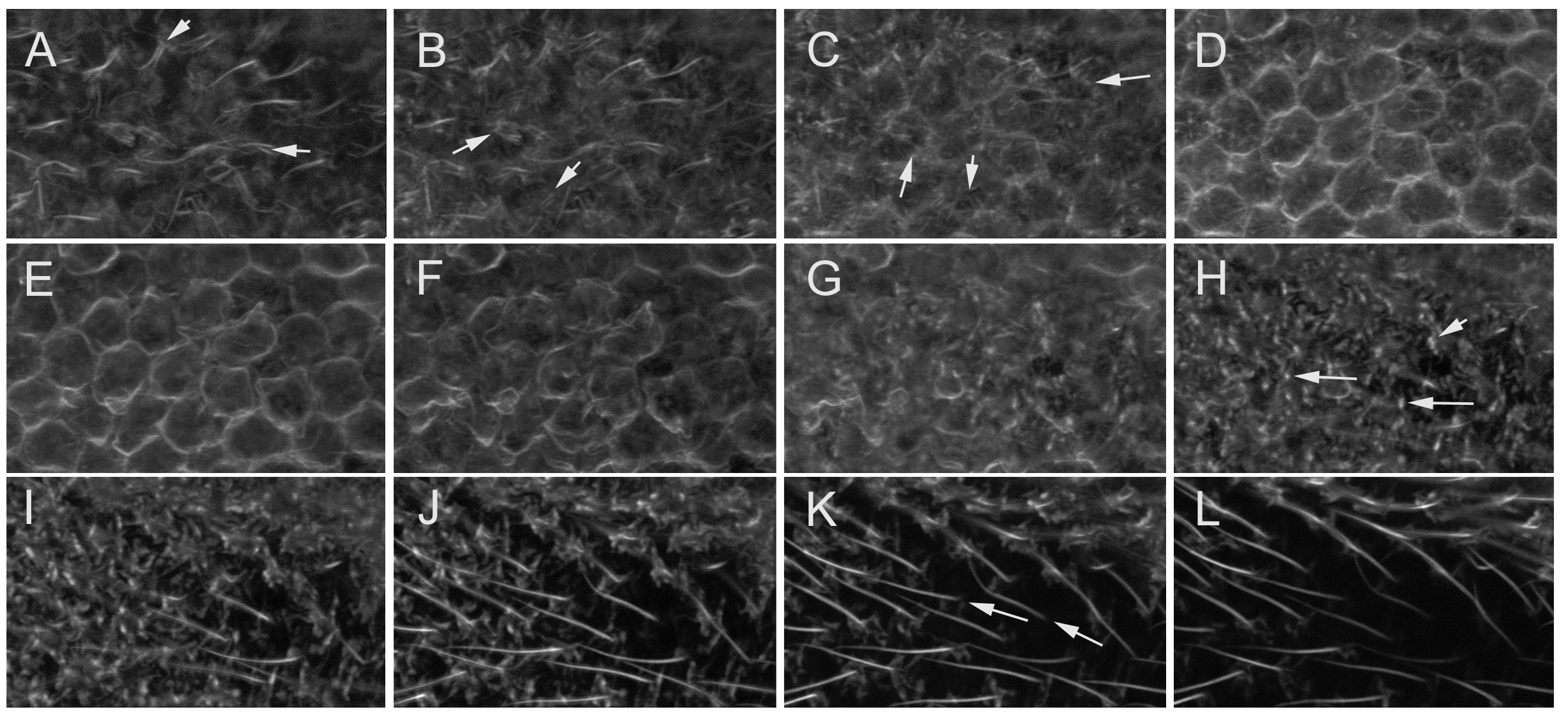

Supplement: S4 Fig — Starting at the dorsal surface and moving ventrally images of ap-Gal4 pTub-Gal80 ts /+; UAS-CA-dia/+ pupal wings stained for F-actin. Each image is a maximum projection of 3 optical sections that represent 0.6um along the dorsal/ventral axis. Arrows in A and B point to multiple hairs cells. The arrows in C point to ectopic actin filaments not normally seen in wing cells. The arrows in H point to actin foci in ventral cells found associated with chitin deposition. These are missing from the dorsal cells. The arrows in K point to normal ventral hairs. (TIF) [file pone.0115623.s004.tif]

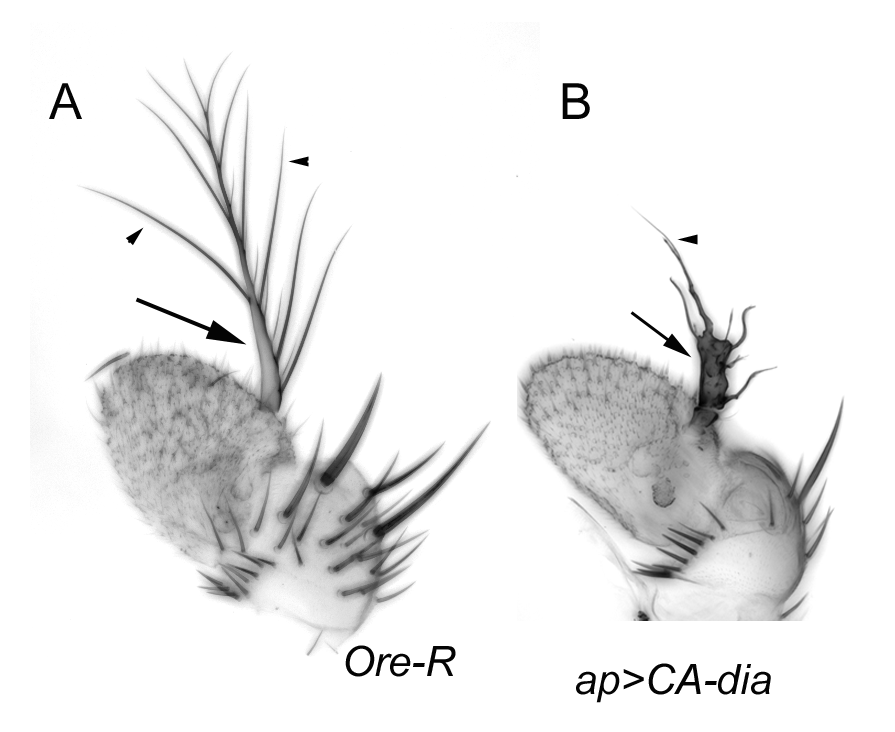

Supplement: S5 Fig — Oregon R (A) and ap>CA-dia (B) arista are shown. The arrow points to the central core which is grossly short and fat in ap>CA-dia. The arrowheads point to laterals which are short and deformed in ap>CA-dia. (TIF) [file pone.0115623.s005.tif]

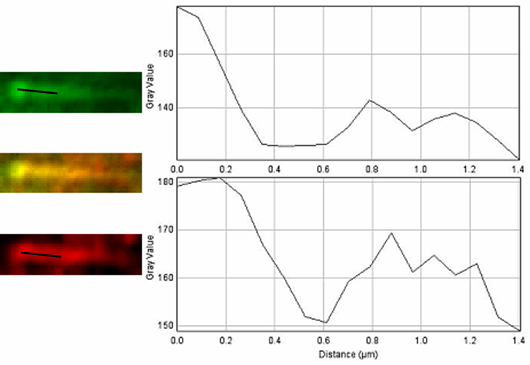

Supplement: S6 Fig — The relative intensity of immunostaining for Dia-GFP (green) and Mwh (red) along the proximal part of a growing hair is shown. The presence of substantial Mwh at the cell periphery prevented us from extending the line of measurement to the full length of the hair. (TIF) [file pone.0115623.s006.tif]

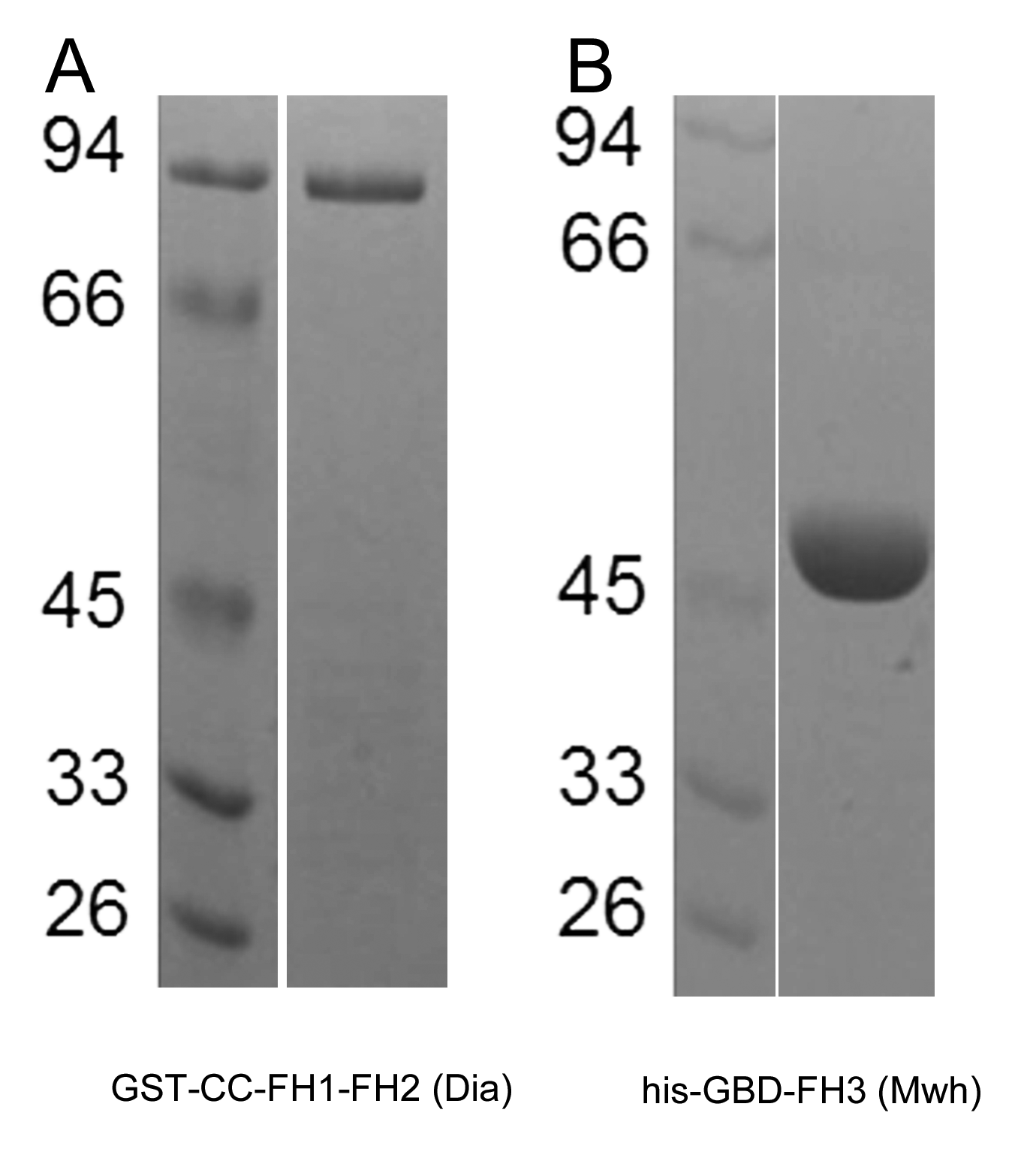

Supplement: S7 Fig — The purified proteins were examined by PAGE and western blotting to confirm their purity. The GST-CC-FH1-FH2 protein migrates slightly faster than the 94 kd marker and the his-GBD-FH3 protein migrates at approximately 45kd. (TIF) [file pone.0115623.s007.tif]
